# Cyclo­hexane-1,3-diyl bis­(*N*-phenyl­carbamate)

**DOI:** 10.1107/S1600536810033817

**Published:** 2010-08-28

**Authors:** Raza Murad Ghalib, Othman Sulaiman, Sayed Hasan Mehdi, Jia Hao Goh, Hoong-Kun Fun

**Affiliations:** a School of Industrial Technology, Universiti Sains Malaysia, 11800 USM, Penang, Malaysia; b X-ray Crystallography Unit, School of Physics, Universiti Sains Malaysia, 11800 USM, Penang, Malaysia

## Abstract

The asymmetric unit of the title compound, C_20_H_22_N_2_O_4_, comprises two crystallographically independent mol­ecules (*A* and *B*) with slightly different geometries. The dihedral angle between the two terminal phenyl rings is 61.7 (1)° in mol­ecule *A* and 29.6 (1)° in *B*. The cyclo­hexane rings adopt chair conformations. In the crystal packing, inter­molecular N—H⋯O hydrogen bonds inter­connect adjacent mol­ecules into a ladder-like structure along the *c* axis incorporating *R*_2_^2^(20) ring motifs. The crystal packing is further stabilized by weak inter­molecular C—H⋯π inter­actions.

## Related literature

For general background and the synthesis of carbamates, see: Banerjee *et al.* (1978 ▶); Ghalib *et al.* (2010 ▶); Graia *et al.* (2009 ▶); Ibuka *et al.* (1985 ▶); Lapidus *et al.* (1987 ▶); Loev & Kormendy (1963 ▶); Niu *et al.* (2007 ▶). For ring conformation and puckering analysis, see: Cremer & Pople (1975 ▶). For graph-set motifs, see: Bernstein *et al.* (1995 ▶). For a related structure, see: Ghalib *et al.* (2010 ▶). For the stability of the temperature controller used in the data collection, see: Cosier & Glazer (1986 ▶).
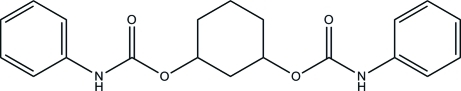


## Experimental

### 

#### Crystal data

C_20_H_22_N_2_O_4_
*M* *_r_* = 354.40
Monoclinic, 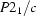

*a* = 38.356 (7) Å
*b* = 9.5453 (16) Å
*c* = 9.8472 (16) Å
β = 91.034 (3)°
*V* = 3604.7 (10) Å^3^
*Z* = 8
Mo *K*α radiation
μ = 0.09 mm^−1^
*T* = 100 K
0.55 × 0.15 × 0.13 mm

#### Data collection

Bruker APEXII DUO CCD area-detector diffractometer
Absorption correction: multi-scan (*SADABS*; Bruker, 2009 ▶) *T*_min_ = 0.951, *T*_max_ = 0.988
30037 measured reflections
10472 independent reflections
6630 reflections with *I* > 2σ(*I*)
*R*_int_ = 0.057

#### Refinement

*R*[*F* ^2^ > 2σ(*F* ^2^)] = 0.064
*wR*(*F* ^2^) = 0.180
*S* = 1.08
10472 reflections
485 parameters
H atoms treated by a mixture of independent and constrained refinement
Δρ_max_ = 0.33 e Å^−3^
Δρ_min_ = −0.25 e Å^−3^

### 

Data collection: *APEX2* (Bruker, 2009 ▶); cell refinement: *SAINT* (Bruker, 2009 ▶); data reduction: *SAINT*; program(s) used to solve structure: *SHELXTL* (Sheldrick, 2008 ▶); program(s) used to refine structure: *SHELXTL*; molecular graphics: *SHELXTL*; software used to prepare material for publication: *SHELXTL* and *PLATON* (Spek, 2009 ▶).

## Supplementary Material

Crystal structure: contains datablocks global, I. DOI: 10.1107/S1600536810033817/ci5161sup1.cif

Structure factors: contains datablocks I. DOI: 10.1107/S1600536810033817/ci5161Isup2.hkl

Additional supplementary materials:  crystallographic information; 3D view; checkCIF report

## Figures and Tables

**Table 1:** Hydrogen-bond geometry (Å, °) *Cg*1 and *Cg*2 are the centroids of C15*A*–C20*A* and C1*B*–C6*B* phenyl rings, respectively.

*D*—H⋯*A*	*D*—H	H⋯*A*	*D*⋯*A*	*D*—H⋯*A*
N1*A*—H1*NA*⋯O1*A*^i^	0.83 (2)	2.13 (2)	2.953 (2)	174 (2)
N2*A*—H2*NA*⋯O4*A*^i^	0.91 (3)	2.09 (3)	2.934 (2)	154 (2)
N1*B*—H1*NB*⋯O1*B*^ii^	0.93 (3)	2.10 (3)	2.920 (2)	147 (2)
N2*B*—H2*NB*⋯O4*B*^ii^	0.88 (3)	2.02 (3)	2.874 (2)	163 (3)
C13*A*—H13*B*⋯*Cg*1^iii^	0.97	2.86	3.734 (2)	151
C13*B*—H13*C*⋯*Cg*2^iv^	0.97	2.86	3.732 (2)	150

Symmetry codes: (i) 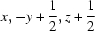; (ii) 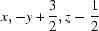; (iii) 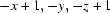; (iv) 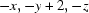.

**Fig. 1.**
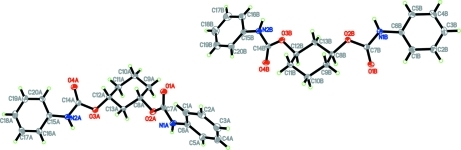
The asymmetric unit of the title compound, showing 50% probability displacement ellipsoids for non-H atoms and the atom-numbering scheme.

**Fig. 2.**
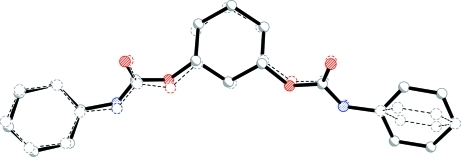
Fit of molecule A (dashed lines) on molecule B (solid lines). H atoms have been omitted for clarity.

**Fig. 3.**
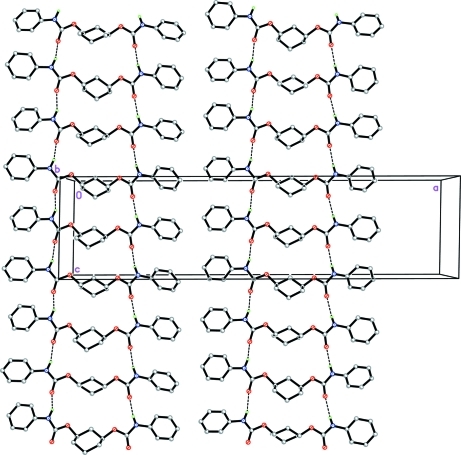
The crystal structure of the title compound, viewed along the b axis, showing molecular chains along the c axis. H atoms not involved in hydrogen bonds (dashed lines) have been omitted for clarity.

## supplementary crystallographic information

### Comment

Carbamates are a well-known class of organic compounds. They can be prepared by nickel-catalyzed coupling of CO_2_ and amines (Niu *et al.*, 2007), by stirring of alcohols including steroids as well as primary and secondary alcohols, polyols, phenols with sodium cyanate and trifluoroacetic acid (Loev & Kormendy, 1963), by carbonylation of aromatic nitro compounds (Lapidus *et al.*, 1987), by the reaction of isocyanates with alcohols in presence of Lewis acid (Ibuka *et al.*, 1985) and by the reaction of an amine and an alcohol with phosgene. Phytosterol, β-sitosterol, stigmasterol, cholesterol, cyclohexanol and α-terpineol react with phenyl isocyanate to give carbamates (Banerjee *et al.*, 1978; Graia *et al.*, 2009; Ghalib *et al.*, 2010). In the present work, the title compound has been synthesized by the reaction of cyclohexane-1,3-diol with phenylisocyanate in the presence of catalytic amount of HCl in chloroform solvent.

The asymmetric unit of the title compound comprises of two crystallographically independent molecules, designated *A* and *B* (Fig. 1). The orientation of the C1–C6 phenyl ring with respect to the rest of the molecule is different in *A* and *B*, as shown in the superposition of the non-H atoms of molecules *A* and *B* (Fig. 2) using *XP* in *SHELXTL* (Sheldrick, 2008); the r.m.s. deviation is 0.474 Å.

In each molecule, the cyclohexane ring (C8-C13) adopts a chair conformation; the puckering parameters are Q = 0.566 (2) Å, θ = 176.6 (2)°, φ = 328 (4)° for molecule *A* and Q = 0.566 (2) Å, θ = 176.2 (2)°, φ = 283 (4)° for molecule *B*. The dihedral angle between the C1–C6 and C15–C20 phenyl rings is 61.7 (1)° in molecule *A* and 29.6 (1)° in *B*. The geometric parameters are consistent with a related structure (Ghalib *et al.*, 2010).

In the crystal packing, intermolecular N1A—H1NA···O1A, N2A—H2NA···O4A, N1B—H1NB···O1B, N2B—H2NB···O4B hydrogen bonds (Table 1) link adjacent molecules into one-dimensional chains incorporating *R*^2^_2_(20) ring motifs (Bernstein *et al.*, 1995) along the *c* axis (Fig. 3). Further stabilization of the crystal packing is provided by weak intermolecular C13A—H13B···*Cg*1 and C13B—H13C···*Cg*2 interactions (Table 1) where *Cg*1 and *Cg*2 are the centroids of C15A-C20A and C1B-C6B phenyl rings, respectively.

### Experimental

A mixture of cyclohexane-1,3-diol (1.005 ml) and phenyl isocyanate (2.174 ml) in a 1:2 molar ratio was stirred in chloroform for 30 minutes in the presence of catalytic amount of HCl. The reaction mixture was dried with rota vapor at low pressure and then crystallized in a 1:1 mixture of chloroform and alcohol to afford colourless needle-like single crystals (yield: 2.60 g, m.p. 489.2 K). The melting point was taken using a Thermo Fisher digital melting point apparatus of IA9000 series and is uncorrected

### Refinement

N-bound H atoms were located in a difference Fourier map and allowed refined freely [range of N—H = 0.83 (2)–0.92 (3) Å]. The remaining H atoms were placed in their calculated positions, with C–H = 0.93–0.98 Å, and refined using a riding model, with *U*_iso_(H) = 1.2*U*_eq_(C).

### Crystal data

**Table:**

C_20_H_22_N_2_O_4_	*F*(000) = 1504
*M**_r_* = 354.40	*D*_x_ = 1.306 Mg m^−^^3^
Monoclinic, *P*2_1_/*c*	Mo *K*α radiation, λ = 0.71073 Å
Hall symbol: -P 2ybc	Cell parameters from 3229 reflections
*a* = 38.356 (7) Å	θ = 3.0–32.1°
*b* = 9.5453 (16) Å	µ = 0.09 mm^−^^1^
*c* = 9.8472 (16) Å	*T* = 100 K
β = 91.034 (3)°	Needle, colourless
*V* = 3604.7 (10) Å^3^	0.55 × 0.15 × 0.13 mm
*Z* = 8	

### Data collection

**Table:**

Bruker APEXII DUO CCD area-detector diffractometer	10472 independent reflections
Radiation source: fine-focus sealed tube	6630 reflections with *I* > 2σ(*I*)
graphite	*R*_int_ = 0.057
φ and ω scans	θ_max_ = 30.0°, θ_min_ = 2.7°
Absorption correction: multi-scan (*SADABS*; Bruker, 2009)	*h* = −51→53
*T*_min_ = 0.951, *T*_max_ = 0.988	*k* = −11→13
30037 measured reflections	*l* = −13→13

### Refinement

**Table:**

Refinement on *F*^2^	Primary atom site location: structure-invariant direct methods
Least-squares matrix: full	Secondary atom site location: difference Fourier map
*R*[*F*^2^ > 2σ(*F*^2^)] = 0.064	Hydrogen site location: inferred from neighbouring sites
*wR*(*F*^2^) = 0.180	H atoms treated by a mixture of independent and constrained refinement
*S* = 1.08	*w* = 1/[σ^2^(*F*_o_^2^) + (0.0587*P*)^2^ + 2.1169*P*] where *P* = (*F*_o_^2^ + 2*F*_c_^2^)/3
10472 reflections	(Δ/σ)_max_ = 0.001
485 parameters	Δρ_max_ = 0.33 e Å^−^^3^
0 restraints	Δρ_min_ = −0.25 e Å^−^^3^

### Special details

**Table:**

Experimental. The crystal was placed in the cold stream of an Oxford Cryosystems Cobra open-flow nitrogen cryostat (Cosier & Glazer, 1986) operating at 100.0 (1)K.
Geometry. All esds (except the esd in the dihedral angle between two l.s. planes) are estimated using the full covariance matrix. The cell esds are taken into account individually in the estimation of esds in distances, angles and torsion angles; correlations between esds in cell parameters are only used when they are defined by crystal symmetry. An approximate (isotropic) treatment of cell esds is used for estimating esds involving l.s. planes.
Refinement. Refinement of F^2^ against ALL reflections. The weighted R-factor wR and goodness of fit S are based on F^2^, conventional R-factors R are based on F, with F set to zero for negative F^2^. The threshold expression of F^2^ > 2sigma(F^2^) is used only for calculating R-factors(gt) etc. and is not relevant to the choice of reflections for refinement. R-factors based on F^2^ are statistically about twice as large as those based on F, and R- factors based on ALL data will be even larger.

### Fractional atomic coordinates and isotropic or equivalent isotropic displacement parameters (Å^2^)

**Table:**

	*x*	*y*	*z*	*U*_iso_*/*U*_eq_	
O1A	0.32324 (4)	0.22226 (16)	0.31570 (14)	0.0210 (3)	
O2A	0.35690 (3)	0.27958 (16)	0.49976 (14)	0.0213 (3)	
O3A	0.47863 (3)	0.24586 (16)	0.53335 (14)	0.0218 (3)	
O4A	0.51876 (4)	0.22687 (17)	0.36729 (14)	0.0259 (3)	
N1A	0.30419 (4)	0.19662 (19)	0.53465 (18)	0.0198 (4)	
N2A	0.53147 (4)	0.17410 (19)	0.59053 (17)	0.0194 (4)	
C1A	0.25950 (5)	0.0743 (2)	0.3965 (2)	0.0215 (4)	
H1AA	0.2760	0.0475	0.3335	0.026*	
C2A	0.22478 (6)	0.0356 (2)	0.3788 (2)	0.0265 (5)	
H2AA	0.2180	−0.0166	0.3031	0.032*	
C3A	0.20014 (6)	0.0740 (3)	0.4726 (2)	0.0290 (5)	
H3AA	0.1769	0.0486	0.4594	0.035*	
C4A	0.21015 (6)	0.1504 (3)	0.5862 (2)	0.0296 (5)	
H4AA	0.1937	0.1747	0.6501	0.036*	
C5A	0.24475 (5)	0.1909 (2)	0.6053 (2)	0.0235 (4)	
H5AA	0.2514	0.2426	0.6816	0.028*	
C6A	0.26940 (5)	0.1536 (2)	0.5095 (2)	0.0194 (4)	
C7A	0.32746 (5)	0.2314 (2)	0.4382 (2)	0.0165 (4)	
C8A	0.38682 (5)	0.3068 (2)	0.4156 (2)	0.0180 (4)	
H8AA	0.3848	0.2531	0.3310	0.022*	
C9A	0.38944 (5)	0.4620 (2)	0.3841 (2)	0.0213 (4)	
H9AA	0.3875	0.5155	0.4673	0.026*	
H9AB	0.3704	0.4891	0.3235	0.026*	
C10A	0.42429 (5)	0.4948 (2)	0.3174 (2)	0.0213 (4)	
H10A	0.4252	0.4481	0.2300	0.026*	
H10B	0.4260	0.5949	0.3017	0.026*	
C11A	0.45518 (5)	0.4471 (2)	0.4063 (2)	0.0210 (4)	
H11A	0.4768	0.4662	0.3603	0.025*	
H11B	0.4554	0.4987	0.4912	0.025*	
C12A	0.45230 (5)	0.2912 (2)	0.4349 (2)	0.0176 (4)	
H12A	0.4548	0.2383	0.3503	0.021*	
C13A	0.41781 (5)	0.2548 (2)	0.4998 (2)	0.0172 (4)	
H13A	0.4171	0.2963	0.5897	0.021*	
H13B	0.4162	0.1539	0.5101	0.021*	
C14A	0.51069 (5)	0.2170 (2)	0.4859 (2)	0.0180 (4)	
C15A	0.56754 (5)	0.1433 (2)	0.5863 (2)	0.0179 (4)	
C16A	0.58223 (5)	0.0713 (2)	0.6959 (2)	0.0206 (4)	
H16A	0.5681	0.0404	0.7656	0.025*	
C17A	0.61787 (6)	0.0451 (2)	0.7023 (2)	0.0255 (5)	
H17A	0.6275	−0.0020	0.7767	0.031*	
C18A	0.63918 (5)	0.0888 (2)	0.5979 (2)	0.0259 (5)	
H18A	0.6631	0.0721	0.6022	0.031*	
C19A	0.62432 (5)	0.1581 (2)	0.4871 (2)	0.0240 (5)	
H19A	0.6384	0.1858	0.4159	0.029*	
C20A	0.58872 (5)	0.1867 (2)	0.4809 (2)	0.0195 (4)	
H20A	0.5791	0.2345	0.4069	0.023*	
O1B	−0.02136 (4)	0.77244 (17)	0.13203 (14)	0.0242 (3)	
O2B	0.01910 (3)	0.75338 (16)	−0.03230 (14)	0.0207 (3)	
O3B	0.14054 (3)	0.71216 (15)	0.00059 (14)	0.0189 (3)	
O4B	0.17604 (4)	0.74059 (17)	0.18627 (14)	0.0242 (3)	
N1B	−0.03341 (4)	0.82633 (18)	−0.09211 (17)	0.0179 (3)	
N2B	0.19386 (4)	0.78876 (19)	−0.03047 (18)	0.0184 (4)	
C1B	−0.09132 (5)	0.8200 (2)	0.0143 (2)	0.0191 (4)	
H1BA	−0.0825	0.7705	0.0888	0.023*	
C2B	−0.12654 (5)	0.8537 (2)	0.0058 (2)	0.0232 (4)	
H2BA	−0.1412	0.8270	0.0754	0.028*	
C3B	−0.14022 (5)	0.9265 (2)	−0.1049 (2)	0.0243 (5)	
H3BA	−0.1639	0.9480	−0.1099	0.029*	
C4B	−0.11823 (5)	0.9667 (2)	−0.2078 (2)	0.0233 (4)	
H4BA	−0.1272	1.0157	−0.2822	0.028*	
C5B	−0.08294 (5)	0.9346 (2)	−0.2011 (2)	0.0195 (4)	
H5BA	−0.0684	0.9622	−0.2707	0.023*	
C6B	−0.06928 (5)	0.8607 (2)	−0.08937 (19)	0.0157 (4)	
C7B	−0.01295 (5)	0.7830 (2)	0.0140 (2)	0.0172 (4)	
C8B	0.04525 (5)	0.7077 (2)	0.06741 (19)	0.0165 (4)	
H8BA	0.0429	0.7617	0.1514	0.020*	
C9B	0.04178 (5)	0.5521 (2)	0.0974 (2)	0.0184 (4)	
H9BA	0.0412	0.4998	0.0129	0.022*	
H9BB	0.0202	0.5348	0.1441	0.022*	
C10B	0.07256 (5)	0.5031 (2)	0.1857 (2)	0.0192 (4)	
H10C	0.0703	0.4034	0.2029	0.023*	
H10D	0.0721	0.5512	0.2724	0.023*	
C11B	0.10739 (5)	0.5314 (2)	0.1179 (2)	0.0194 (4)	
H11C	0.1264	0.5028	0.1780	0.023*	
H11D	0.1088	0.4774	0.0348	0.023*	
C12B	0.11062 (5)	0.6861 (2)	0.08605 (19)	0.0160 (4)	
H12B	0.1130	0.7398	0.1706	0.019*	
C13B	0.07986 (5)	0.7408 (2)	0.0023 (2)	0.0168 (4)	
H13C	0.0821	0.8415	−0.0080	0.020*	
H13D	0.0803	0.6992	−0.0875	0.020*	
C14B	0.17103 (5)	0.7465 (2)	0.0642 (2)	0.0162 (4)	
C15B	0.22895 (5)	0.8287 (2)	−0.00429 (19)	0.0178 (4)	
C16B	0.24314 (6)	0.9297 (2)	−0.0886 (2)	0.0247 (5)	
H16B	0.2294	0.9715	−0.1560	0.030*	
C17B	0.27798 (6)	0.9682 (3)	−0.0719 (3)	0.0342 (6)	
H17B	0.2877	1.0338	−0.1299	0.041*	
C18B	0.29811 (6)	0.9090 (3)	0.0306 (3)	0.0374 (6)	
H18B	0.3212	0.9362	0.0428	0.045*	
C19B	0.28398 (6)	0.8100 (3)	0.1147 (3)	0.0337 (6)	
H19B	0.2976	0.7708	0.1839	0.040*	
C20B	0.24941 (5)	0.7677 (2)	0.0972 (2)	0.0251 (5)	
H20B	0.2401	0.6992	0.1532	0.030*	
H1NA	0.3111 (6)	0.219 (2)	0.612 (2)	0.014 (6)*	
H2NA	0.5227 (7)	0.181 (3)	0.676 (3)	0.048 (8)*	
H1NB	−0.0234 (7)	0.824 (3)	−0.177 (3)	0.038 (7)*	
H2NB	0.1855 (6)	0.795 (3)	−0.114 (3)	0.031 (7)*	

### Atomic displacement parameters (Å^2^)

**Table:**

	*U*^11^	*U*^22^	*U*^33^	*U*^12^	*U*^13^	*U*^23^
O1A	0.0194 (7)	0.0295 (8)	0.0140 (7)	−0.0001 (6)	−0.0018 (5)	0.0010 (6)
O2A	0.0154 (6)	0.0328 (8)	0.0156 (7)	−0.0043 (6)	0.0008 (5)	−0.0037 (6)
O3A	0.0153 (6)	0.0357 (9)	0.0142 (7)	0.0044 (6)	−0.0003 (5)	0.0007 (6)
O4A	0.0215 (7)	0.0415 (9)	0.0147 (7)	0.0057 (7)	0.0007 (6)	0.0019 (7)
N1A	0.0174 (8)	0.0301 (10)	0.0118 (8)	−0.0032 (7)	−0.0010 (6)	−0.0028 (7)
N2A	0.0176 (8)	0.0271 (9)	0.0136 (8)	0.0022 (7)	−0.0005 (6)	0.0017 (7)
C1A	0.0207 (9)	0.0231 (10)	0.0206 (10)	−0.0015 (8)	−0.0035 (8)	0.0004 (8)
C2A	0.0260 (11)	0.0282 (12)	0.0251 (11)	−0.0075 (9)	−0.0090 (9)	0.0016 (9)
C3A	0.0187 (10)	0.0334 (13)	0.0346 (13)	−0.0067 (9)	−0.0050 (9)	0.0081 (10)
C4A	0.0204 (10)	0.0326 (12)	0.0361 (13)	−0.0001 (9)	0.0058 (9)	0.0038 (10)
C5A	0.0204 (10)	0.0276 (11)	0.0225 (11)	−0.0009 (8)	0.0020 (8)	−0.0022 (9)
C6A	0.0166 (9)	0.0199 (10)	0.0214 (10)	−0.0024 (7)	−0.0017 (8)	0.0027 (8)
C7A	0.0155 (9)	0.0179 (9)	0.0161 (10)	0.0012 (7)	−0.0032 (7)	−0.0013 (7)
C8A	0.0158 (8)	0.0230 (10)	0.0154 (9)	−0.0015 (7)	0.0021 (7)	−0.0004 (8)
C9A	0.0209 (9)	0.0215 (10)	0.0215 (10)	0.0025 (8)	−0.0017 (8)	0.0009 (8)
C10A	0.0281 (10)	0.0168 (10)	0.0190 (10)	−0.0029 (8)	−0.0010 (8)	0.0000 (8)
C11A	0.0204 (9)	0.0241 (11)	0.0185 (10)	−0.0056 (8)	0.0028 (8)	−0.0012 (8)
C12A	0.0157 (9)	0.0240 (10)	0.0131 (9)	0.0014 (7)	−0.0025 (7)	−0.0007 (8)
C13A	0.0169 (9)	0.0204 (10)	0.0143 (9)	0.0000 (7)	0.0014 (7)	0.0019 (7)
C14A	0.0157 (9)	0.0222 (10)	0.0161 (9)	0.0010 (7)	0.0006 (7)	−0.0013 (8)
C15A	0.0186 (9)	0.0178 (9)	0.0173 (10)	0.0013 (7)	−0.0015 (7)	−0.0030 (8)
C16A	0.0244 (10)	0.0201 (10)	0.0172 (10)	0.0033 (8)	−0.0023 (8)	−0.0026 (8)
C17A	0.0276 (11)	0.0243 (11)	0.0243 (11)	0.0062 (9)	−0.0076 (9)	−0.0041 (9)
C18A	0.0184 (10)	0.0280 (12)	0.0310 (12)	0.0037 (8)	−0.0049 (9)	−0.0061 (10)
C19A	0.0210 (10)	0.0222 (11)	0.0288 (12)	0.0001 (8)	0.0021 (8)	−0.0021 (9)
C20A	0.0205 (9)	0.0199 (10)	0.0181 (10)	0.0015 (8)	−0.0001 (8)	0.0010 (8)
O1B	0.0218 (7)	0.0381 (9)	0.0128 (7)	0.0066 (6)	−0.0001 (6)	0.0021 (6)
O2B	0.0144 (6)	0.0333 (8)	0.0145 (7)	0.0035 (6)	−0.0014 (5)	0.0018 (6)
O3B	0.0142 (6)	0.0283 (8)	0.0142 (7)	−0.0040 (6)	0.0004 (5)	−0.0019 (6)
O4B	0.0204 (7)	0.0407 (9)	0.0115 (7)	−0.0029 (6)	−0.0017 (5)	0.0021 (6)
N1B	0.0164 (8)	0.0256 (9)	0.0116 (8)	0.0022 (7)	0.0000 (6)	0.0010 (7)
N2B	0.0148 (7)	0.0281 (9)	0.0123 (8)	−0.0021 (7)	−0.0012 (6)	0.0010 (7)
C1B	0.0202 (9)	0.0201 (10)	0.0170 (10)	0.0005 (8)	−0.0010 (8)	−0.0016 (8)
C2B	0.0197 (9)	0.0252 (11)	0.0249 (11)	0.0004 (8)	0.0025 (8)	−0.0029 (9)
C3B	0.0178 (9)	0.0238 (11)	0.0312 (12)	0.0037 (8)	−0.0046 (8)	−0.0030 (9)
C4B	0.0252 (10)	0.0210 (10)	0.0233 (11)	0.0038 (8)	−0.0083 (8)	−0.0021 (8)
C5B	0.0244 (10)	0.0196 (10)	0.0145 (9)	0.0019 (8)	−0.0023 (8)	0.0003 (8)
C6B	0.0158 (8)	0.0174 (9)	0.0139 (9)	0.0009 (7)	−0.0028 (7)	−0.0034 (7)
C7B	0.0174 (9)	0.0195 (10)	0.0146 (9)	0.0001 (7)	−0.0002 (7)	−0.0018 (8)
C8B	0.0151 (8)	0.0223 (10)	0.0121 (9)	0.0012 (7)	−0.0020 (7)	0.0001 (7)
C9B	0.0174 (9)	0.0223 (10)	0.0154 (9)	−0.0038 (7)	−0.0008 (7)	−0.0009 (8)
C10B	0.0222 (9)	0.0184 (10)	0.0169 (10)	−0.0032 (8)	−0.0003 (8)	0.0015 (8)
C11B	0.0197 (9)	0.0204 (10)	0.0179 (10)	0.0027 (8)	−0.0010 (7)	0.0017 (8)
C12B	0.0136 (8)	0.0210 (10)	0.0134 (9)	−0.0011 (7)	0.0007 (7)	0.0002 (7)
C13B	0.0162 (9)	0.0183 (10)	0.0159 (10)	−0.0013 (7)	−0.0022 (7)	0.0019 (7)
C14B	0.0145 (8)	0.0202 (10)	0.0138 (9)	0.0001 (7)	−0.0002 (7)	0.0001 (7)
C15B	0.0163 (9)	0.0235 (10)	0.0137 (9)	−0.0022 (8)	0.0014 (7)	−0.0048 (8)
C16B	0.0256 (10)	0.0243 (11)	0.0242 (11)	−0.0037 (9)	0.0001 (8)	0.0014 (9)
C17B	0.0333 (12)	0.0316 (13)	0.0379 (14)	−0.0133 (10)	0.0046 (11)	−0.0009 (11)
C18B	0.0209 (11)	0.0450 (15)	0.0462 (16)	−0.0111 (10)	−0.0010 (10)	−0.0063 (13)
C19B	0.0217 (11)	0.0485 (16)	0.0306 (13)	0.0002 (10)	−0.0066 (9)	−0.0002 (11)
C20B	0.0181 (9)	0.0359 (12)	0.0212 (11)	−0.0019 (9)	−0.0020 (8)	0.0044 (9)

### Geometric parameters (Å, °)

**Table:**

O1A—C7A	1.218 (2)	O1B—C7B	1.216 (2)
O2A—C7A	1.352 (2)	O2B—C7B	1.349 (2)
O2A—C8A	1.451 (2)	O2B—C8B	1.457 (2)
O3A—C14A	1.352 (2)	O3B—C14B	1.357 (2)
O3A—C12A	1.453 (2)	O3B—C12B	1.457 (2)
O4A—C14A	1.218 (2)	O4B—C14B	1.215 (2)
N1A—C7A	1.356 (2)	N1B—C7B	1.360 (3)
N1A—C6A	1.414 (3)	N1B—C6B	1.415 (2)
N1A—H1NA	0.83 (2)	N1B—H1NB	0.92 (3)
N2A—C14A	1.354 (3)	N2B—C14B	1.352 (2)
N2A—C15A	1.416 (2)	N2B—C15B	1.418 (2)
N2A—H2NA	0.92 (3)	N2B—H2NB	0.88 (3)
C1A—C2A	1.390 (3)	C1B—C2B	1.390 (3)
C1A—C6A	1.392 (3)	C1B—C6B	1.393 (2)
C1A—H1AA	0.93	C1B—H1BA	0.93
C2A—C3A	1.383 (3)	C2B—C3B	1.387 (3)
C2A—H2AA	0.93	C2B—H2BA	0.93
C3A—C4A	1.383 (3)	C3B—C4B	1.384 (3)
C3A—H3AA	0.93	C3B—H3BA	0.93
C4A—C5A	1.392 (3)	C4B—C5B	1.388 (3)
C4A—H4AA	0.93	C4B—H4BA	0.93
C5A—C6A	1.394 (3)	C5B—C6B	1.400 (3)
C5A—H5AA	0.93	C5B—H5BA	0.93
C8A—C9A	1.517 (3)	C8B—C13B	1.518 (2)
C8A—C13A	1.520 (3)	C8B—C9B	1.520 (3)
C8A—H8AA	0.98	C8B—H8BA	0.98
C9A—C10A	1.532 (3)	C9B—C10B	1.527 (3)
C9A—H9AA	0.97	C9B—H9BA	0.97
C9A—H9AB	0.97	C9B—H9BB	0.97
C10A—C11A	1.530 (3)	C10B—C11B	1.528 (3)
C10A—H10A	0.97	C10B—H10C	0.97
C10A—H10B	0.97	C10B—H10D	0.97
C11A—C12A	1.519 (3)	C11B—C12B	1.515 (3)
C11A—H11A	0.97	C11B—H11C	0.97
C11A—H11B	0.97	C11B—H11D	0.97
C12A—C13A	1.520 (2)	C12B—C13B	1.520 (3)
C12A—H12A	0.98	C12B—H12B	0.98
C13A—H13A	0.97	C13B—H13C	0.97
C13A—H13B	0.97	C13B—H13D	0.97
C15A—C16A	1.390 (3)	C15B—C20B	1.388 (3)
C15A—C20A	1.393 (3)	C15B—C16B	1.390 (3)
C16A—C17A	1.390 (3)	C16B—C17B	1.393 (3)
C16A—H16A	0.93	C16B—H16B	0.93
C17A—C18A	1.389 (3)	C17B—C18B	1.381 (4)
C17A—H17A	0.93	C17B—H17B	0.93
C18A—C19A	1.390 (3)	C18B—C19B	1.375 (3)
C18A—H18A	0.93	C18B—H18B	0.93
C19A—C20A	1.393 (3)	C19B—C20B	1.394 (3)
C19A—H19A	0.93	C19B—H19B	0.93
C20A—H20A	0.93	C20B—H20B	0.93
			
C7A—O2A—C8A	117.89 (15)	C7B—O2B—C8B	117.06 (14)
C14A—O3A—C12A	117.13 (15)	C14B—O3B—C12B	117.12 (14)
C7A—N1A—C6A	125.41 (18)	C7B—N1B—C6B	127.13 (16)
C7A—N1A—H1NA	112.3 (15)	C7B—N1B—H1NB	116.4 (17)
C6A—N1A—H1NA	121.2 (15)	C6B—N1B—H1NB	116.2 (17)
C14A—N2A—C15A	127.07 (16)	C14B—N2B—C15B	125.39 (18)
C14A—N2A—H2NA	117.3 (18)	C14B—N2B—H2NB	115.7 (16)
C15A—N2A—H2NA	114.6 (18)	C15B—N2B—H2NB	118.8 (16)
C2A—C1A—C6A	119.43 (19)	C2B—C1B—C6B	119.5 (2)
C2A—C1A—H1AA	120.3	C2B—C1B—H1BA	120.2
C6A—C1A—H1AA	120.3	C6B—C1B—H1BA	120.2
C3A—C2A—C1A	120.7 (2)	C3B—C2B—C1B	121.13 (19)
C3A—C2A—H2AA	119.6	C3B—C2B—H2BA	119.4
C1A—C2A—H2AA	119.6	C1B—C2B—H2BA	119.4
C2A—C3A—C4A	119.8 (2)	C4B—C3B—C2B	119.13 (19)
C2A—C3A—H3AA	120.1	C4B—C3B—H3BA	120.4
C4A—C3A—H3AA	120.1	C2B—C3B—H3BA	120.4
C3A—C4A—C5A	120.4 (2)	C3B—C4B—C5B	120.7 (2)
C3A—C4A—H4AA	119.8	C3B—C4B—H4BA	119.6
C5A—C4A—H4AA	119.8	C5B—C4B—H4BA	119.6
C4A—C5A—C6A	119.6 (2)	C4B—C5B—C6B	119.94 (18)
C4A—C5A—H5AA	120.2	C4B—C5B—H5BA	120.0
C6A—C5A—H5AA	120.2	C6B—C5B—H5BA	120.0
C1A—C6A—C5A	120.07 (19)	C1B—C6B—C5B	119.55 (18)
C1A—C6A—N1A	122.74 (18)	C1B—C6B—N1B	123.52 (18)
C5A—C6A—N1A	117.16 (19)	C5B—C6B—N1B	116.87 (16)
O1A—C7A—O2A	124.28 (17)	O1B—C7B—O2B	124.40 (19)
O1A—C7A—N1A	126.79 (19)	O1B—C7B—N1B	126.90 (18)
O2A—C7A—N1A	108.94 (17)	O2B—C7B—N1B	108.70 (15)
O2A—C8A—C9A	110.34 (15)	O2B—C8B—C13B	104.44 (15)
O2A—C8A—C13A	104.39 (15)	O2B—C8B—C9B	111.20 (16)
C9A—C8A—C13A	112.13 (17)	C13B—C8B—C9B	111.45 (15)
O2A—C8A—H8AA	110.0	O2B—C8B—H8BA	109.9
C9A—C8A—H8AA	110.0	C13B—C8B—H8BA	109.9
C13A—C8A—H8AA	110.0	C9B—C8B—H8BA	109.9
C8A—C9A—C10A	110.43 (16)	C8B—C9B—C10B	109.91 (16)
C8A—C9A—H9AA	109.6	C8B—C9B—H9BA	109.7
C10A—C9A—H9AA	109.6	C10B—C9B—H9BA	109.7
C8A—C9A—H9AB	109.6	C8B—C9B—H9BB	109.7
C10A—C9A—H9AB	109.6	C10B—C9B—H9BB	109.7
H9AA—C9A—H9AB	108.1	H9BA—C9B—H9BB	108.2
C11A—C10A—C9A	111.48 (16)	C9B—C10B—C11B	111.69 (16)
C11A—C10A—H10A	109.3	C9B—C10B—H10C	109.3
C9A—C10A—H10A	109.3	C11B—C10B—H10C	109.3
C11A—C10A—H10B	109.3	C9B—C10B—H10D	109.3
C9A—C10A—H10B	109.3	C11B—C10B—H10D	109.3
H10A—C10A—H10B	108.0	H10C—C10B—H10D	107.9
C12A—C11A—C10A	109.87 (17)	C12B—C11B—C10B	109.78 (16)
C12A—C11A—H11A	109.7	C12B—C11B—H11C	109.7
C10A—C11A—H11A	109.7	C10B—C11B—H11C	109.7
C12A—C11A—H11B	109.7	C12B—C11B—H11D	109.7
C10A—C11A—H11B	109.7	C10B—C11B—H11D	109.7
H11A—C11A—H11B	108.2	H11C—C11B—H11D	108.2
O3A—C12A—C11A	111.31 (16)	O3B—C12B—C11B	110.74 (15)
O3A—C12A—C13A	104.54 (15)	O3B—C12B—C13B	103.88 (14)
C11A—C12A—C13A	111.63 (16)	C11B—C12B—C13B	112.44 (16)
O3A—C12A—H12A	109.7	O3B—C12B—H12B	109.9
C11A—C12A—H12A	109.7	C11B—C12B—H12B	109.9
C13A—C12A—H12A	109.7	C13B—C12B—H12B	109.9
C12A—C13A—C8A	111.94 (16)	C8B—C13B—C12B	112.05 (16)
C12A—C13A—H13A	109.2	C8B—C13B—H13C	109.2
C8A—C13A—H13A	109.2	C12B—C13B—H13C	109.2
C12A—C13A—H13B	109.2	C8B—C13B—H13D	109.2
C8A—C13A—H13B	109.2	C12B—C13B—H13D	109.2
H13A—C13A—H13B	107.9	H13C—C13B—H13D	107.9
O4A—C14A—O3A	124.35 (19)	O4B—C14B—N2B	127.10 (19)
O4A—C14A—N2A	126.63 (18)	O4B—C14B—O3B	124.41 (16)
O3A—C14A—N2A	109.01 (16)	N2B—C14B—O3B	108.48 (16)
C16A—C15A—C20A	119.49 (18)	C20B—C15B—C16B	119.92 (19)
C16A—C15A—N2A	117.55 (17)	C20B—C15B—N2B	122.74 (18)
C20A—C15A—N2A	122.91 (19)	C16B—C15B—N2B	117.30 (19)
C17A—C16A—C15A	120.56 (19)	C15B—C16B—C17B	119.9 (2)
C17A—C16A—H16A	119.7	C15B—C16B—H16B	120.1
C15A—C16A—H16A	119.7	C17B—C16B—H16B	120.1
C18A—C17A—C16A	120.3 (2)	C18B—C17B—C16B	120.0 (2)
C18A—C17A—H17A	119.9	C18B—C17B—H17B	120.0
C16A—C17A—H17A	119.9	C16B—C17B—H17B	120.0
C17A—C18A—C19A	119.06 (19)	C19B—C18B—C17B	120.0 (2)
C17A—C18A—H18A	120.5	C19B—C18B—H18B	120.0
C19A—C18A—H18A	120.5	C17B—C18B—H18B	120.0
C18A—C19A—C20A	121.02 (19)	C18B—C19B—C20B	120.6 (2)
C18A—C19A—H19A	119.5	C18B—C19B—H19B	119.7
C20A—C19A—H19A	119.5	C20B—C19B—H19B	119.7
C15A—C20A—C19A	119.6 (2)	C15B—C20B—C19B	119.5 (2)
C15A—C20A—H20A	120.2	C15B—C20B—H20B	120.3
C19A—C20A—H20A	120.2	C19B—C20B—H20B	120.3
			
C6A—C1A—C2A—C3A	−0.6 (3)	C6B—C1B—C2B—C3B	0.5 (3)
C1A—C2A—C3A—C4A	−0.7 (3)	C1B—C2B—C3B—C4B	−0.4 (3)
C2A—C3A—C4A—C5A	1.2 (4)	C2B—C3B—C4B—C5B	0.1 (3)
C3A—C4A—C5A—C6A	−0.3 (3)	C3B—C4B—C5B—C6B	0.1 (3)
C2A—C1A—C6A—C5A	1.5 (3)	C2B—C1B—C6B—C5B	−0.3 (3)
C2A—C1A—C6A—N1A	179.8 (2)	C2B—C1B—C6B—N1B	−177.38 (19)
C4A—C5A—C6A—C1A	−1.1 (3)	C4B—C5B—C6B—C1B	0.0 (3)
C4A—C5A—C6A—N1A	−179.5 (2)	C4B—C5B—C6B—N1B	177.30 (18)
C7A—N1A—C6A—C1A	34.4 (3)	C7B—N1B—C6B—C1B	−18.0 (3)
C7A—N1A—C6A—C5A	−147.2 (2)	C7B—N1B—C6B—C5B	164.83 (19)
C8A—O2A—C7A—O1A	−7.5 (3)	C8B—O2B—C7B—O1B	−1.0 (3)
C8A—O2A—C7A—N1A	172.67 (16)	C8B—O2B—C7B—N1B	178.74 (16)
C6A—N1A—C7A—O1A	−5.5 (3)	C6B—N1B—C7B—O1B	−3.8 (3)
C6A—N1A—C7A—O2A	174.33 (18)	C6B—N1B—C7B—O2B	176.52 (18)
C7A—O2A—C8A—C9A	99.4 (2)	C7B—O2B—C8B—C13B	−157.19 (16)
C7A—O2A—C8A—C13A	−139.95 (17)	C7B—O2B—C8B—C9B	82.5 (2)
O2A—C8A—C9A—C10A	169.92 (16)	O2B—C8B—C9B—C10B	171.80 (14)
C13A—C8A—C9A—C10A	54.0 (2)	C13B—C8B—C9B—C10B	55.7 (2)
C8A—C9A—C10A—C11A	−56.4 (2)	C8B—C9B—C10B—C11B	−57.9 (2)
C9A—C10A—C11A—C12A	57.4 (2)	C9B—C10B—C11B—C12B	57.0 (2)
C14A—O3A—C12A—C11A	−83.1 (2)	C14B—O3B—C12B—C11B	−93.5 (2)
C14A—O3A—C12A—C13A	156.25 (17)	C14B—O3B—C12B—C13B	145.54 (16)
C10A—C11A—C12A—O3A	−172.51 (14)	C10B—C11B—C12B—O3B	−170.03 (15)
C10A—C11A—C12A—C13A	−56.1 (2)	C10B—C11B—C12B—C13B	−54.3 (2)
O3A—C12A—C13A—C8A	174.99 (15)	O2B—C8B—C13B—C12B	−174.04 (15)
C11A—C12A—C13A—C8A	54.5 (2)	C9B—C8B—C13B—C12B	−53.9 (2)
O2A—C8A—C13A—C12A	−172.95 (15)	O3B—C12B—C13B—C8B	173.31 (15)
C9A—C8A—C13A—C12A	−53.5 (2)	C11B—C12B—C13B—C8B	53.5 (2)
C12A—O3A—C14A—O4A	0.2 (3)	C15B—N2B—C14B—O4B	3.0 (3)
C12A—O3A—C14A—N2A	−178.81 (16)	C15B—N2B—C14B—O3B	−177.74 (18)
C15A—N2A—C14A—O4A	5.8 (4)	C12B—O3B—C14B—O4B	9.2 (3)
C15A—N2A—C14A—O3A	−175.19 (18)	C12B—O3B—C14B—N2B	−170.07 (16)
C14A—N2A—C15A—C16A	−165.8 (2)	C14B—N2B—C15B—C20B	33.7 (3)
C14A—N2A—C15A—C20A	16.8 (3)	C14B—N2B—C15B—C16B	−148.7 (2)
C20A—C15A—C16A—C17A	1.4 (3)	C20B—C15B—C16B—C17B	0.8 (3)
N2A—C15A—C16A—C17A	−176.11 (19)	N2B—C15B—C16B—C17B	−176.9 (2)
C15A—C16A—C17A—C18A	−1.0 (3)	C15B—C16B—C17B—C18B	−1.9 (4)
C16A—C17A—C18A—C19A	−0.5 (3)	C16B—C17B—C18B—C19B	1.3 (4)
C17A—C18A—C19A—C20A	1.5 (3)	C17B—C18B—C19B—C20B	0.3 (4)
C16A—C15A—C20A—C19A	−0.4 (3)	C16B—C15B—C20B—C19B	0.8 (3)
N2A—C15A—C20A—C19A	176.97 (19)	N2B—C15B—C20B—C19B	178.4 (2)
C18A—C19A—C20A—C15A	−1.1 (3)	C18B—C19B—C20B—C15B	−1.4 (4)

### Hydrogen-bond geometry (Å, °)

**Table:**

Cg1 and Cg2 are the centroids of C15A–C20A and C1B–C6B phenyl rings, respectively.

**Table:**

*D*—H···*A*	*D*—H	H···*A*	*D*···*A*	*D*—H···*A*
N1A—H1NA···O1A^i^	0.83 (2)	2.13 (2)	2.953 (2)	174 (2)
N2A—H2NA···O4A^i^	0.91 (3)	2.09 (3)	2.934 (2)	154 (2)
N1B—H1NB···O1B^ii^	0.93 (3)	2.10 (3)	2.920 (2)	147 (2)
N2B—H2NB···O4B^ii^	0.88 (3)	2.02 (3)	2.874 (2)	163 (3)
C13A—H13B···Cg1^iii^	0.97	2.86	3.734 (2)	151
C13B—H13C···Cg2^iv^	0.97	2.86	3.732 (2)	150

Symmetry codes: (i) *x*, −*y*+1/2, *z*+1/2; (ii) *x*, −*y*+3/2, *z*−1/2; (iii) −*x*+1, −*y*, −*z*+1; (iv) −*x*, −*y*+2, −*z*.
